# Clinicians’ perceptions of the health status of formerly detained immigrants

**DOI:** 10.1186/s12889-022-12967-7

**Published:** 2022-03-23

**Authors:** Kathryn Hampton, Ranit Mishori, Marsha Griffin, Claire Hillier, Elizabeth Pirrotta, N. Ewen Wang

**Affiliations:** 1 Physicians for Human Rights, New York, NY USA; 2 Department of Family Medicine, Georgetown University School of Medicine, Washington, DC, USA; 3 Department of Pediatrics, University of Texas Rio Grande Valley School of Medicine, Edinburg, TX USA; 4 Department of Human Biology, Stanford University, Stanford, California USA; 5 Department of Emergency Medicine, Division of Population Health Research, Stanford University School of Medicine, Stanford, California USA; 6 Department of Emergency Medicine, Division of Pediatric Emergency Medicine, Stanford University School of Medicine, Stanford, California USA

**Keywords:** Immigration Detention, Social determinants of health, Maternal and child health, Chronic and infectious disease epidemiology

## Abstract

**Background:**

In the past decade, the U.S. immigration detention system regularly detained more than 30,000 people per day; in 2019 prior to the pandemic, the daily detention population exceeded 52,000 people. Inhumane detention conditions have been documented by internal government watchdogs, and news media and human rights groups who have observed over-crowding, poor hygiene and sanitation and poor and delayed medical care, as well as verbal, physical and sexual abuse.

**Methods:**

This study surveyed health professionals across the United States who had provided care for immigrants who were recently released from immigration detention to assess clinician perceptions about the adverse health impact of immigration detention on migrant populations based on real-life clinical encounters. There were 150 survey responses, of which 85 clinicians observed medical conditions attributed to detention.

**Results:**

These 85 clinicians reported seeing a combined estimate of 1300 patients with a medical issue related to their time in detention, including patients with delayed access to medical care or medicine in detention, patients with new or acute health conditions such as infection and injury attributed to detention, and patients with worsened chronic or special needs conditions. Clinicians also provided details regarding sentinel cases, categorized into the following themes: Pregnant women, Children, Mentally Ill, COVID-19, and Other serious health issue.

**Conclusions:**

This is the first survey, to our knowledge, of health care professionals treating individuals upon release from detention. Due to the lack of transparency by federal entities and limited access to detainees, this survey serves as a source of credible information about conditions experienced within immigration detention facilities and is a means of corroborating immigrant testimonials and media reports. These findings can help inform policy discussions regarding systematic changes to the delivery of healthcare in detention, quality assurance and transparent reporting.

## Background

For years, news reports, civil society, and human rights groups have documented inhumane conditions in United States (US) immigration detention, characterized by over-crowding, poor hygiene and decreased access to water and sanitation, direct verbal, physical and sexual abuse [1], as well as poor, negligent and delayed medical care [2]. During the Trump administration, conditions reportedly worsened due to a substantial increase in the number of people detained [3], increased duration of detention [4] and policy decisions not to release at-risk populations, such as pregnant people [5] or asylum seekers [6], who would ordinarily have been presumptively released or released after requesting bond [7]. While the Biden administration has reversed some of the policies regarding detention, at the writing of this article, there is another surge at the border, contributing to increasing numbers of asylum seekers, including children, being detained in different types of facilities.

Immigrants can be detained in a number of different types of facilities (Table 1). They each have different governance, infrastructure and health care facilities and protocols that determine access to care. Many immigrants do not know where they were detained, thus making it difficult for physicians to know where to report medical problems in a particular facility. It is also important to note that Customs and Border Protection (CBP), Immigration and Customs Enforcement (ICE) and the Office of Refugee Resettlement (ORR) operate their detention facilities in a non-transparent manner with little external medical oversight. The only individuals who may become aware of worsening medical conditions are the immigrants’ attorneys or physicians treating them once they are released.

**Table 1:** Description of Immigrant Detention facilities

Facility	Governance	Target population	Purpose
United States Customs and Border Protection (CBP)	Department of Homeland Security	All people crossing the border without documentation, including men, women, boys and girls	Processing or intake usually at the border, usually the first point of detention
U.S. Immigration and Customs Enforcement (ICE)^a^	Department of Homeland Security (Including Service Processing Centers, Contract Detention Facilities, Intergovernmental Service Agreements, U.S. Marshals Service Intergovernmental Agreement) [8]	Adults (men and women) or Families (parents and children)	Mandatory detention for certain categories of immigrants while their immigration proceedings pend; ICE has discretion to release on bond or parole, immigration judges may release on bond
State-licensed shelters run by non-profit organizations throughout the country to detain unaccompanied children until sponsors can be identified and screened for reunification	U.S. Department of Health and Human Services; Office of Refugee Resettlement (ORR)	Unaccompanied children	Holding children while locating a family member or other eligible sponsor while their legal case is pending, until the child turns 18

^a^ICE contracts with both local governments and private prison companies, such as the GEO Group, Inc. and Core Civic, to operate the majority of its vast network of facilities [9]

While news reports and other official investigations have documented poor conditions and lapses in medical care, much of this information has not been systematically obtained or published. As a result, it has been difficult to observe trends including the incidence or prevalence of specific conditions, or even to obtain timely details about sentinel events such as deaths. Painstaking efforts to analyze the limited publicly available data through collaboration with legal organizations have resulted in several studies which have shown that deaths in detention are linked with substandard medical care [10, 11], that COVID-19 infection spread more rapidly in immigration detention than in the general U.S. population [12], that COVID-19 prevention and response measures were poorly handled in detention [13], and that release from immigration detention may improve physical and mental health [14]. All of these studies note a dearth of information on the health of people in detention and after their release, as well as the challenges of conducting research because the population is hard to reach and due to lack of government transparency.

Health professionals in the hospital or community setting may see individuals after they are released from federal detention, be it CBP, ICE or ORR detention. In some instances, health professionals have informally shared de-identified information through professional networks and social media groups about the negative health status of some of their patients that they attribute to their time spent in immigration detention. However, we sought to systematically collect health care professionals’ reports and impressions about the impact of immigration detention on their patients’ health and well-being. We also sought to identify reporting practices of health care professionals for these incidents.

## Methods

### Survey design

The authors developed a survey directed at clinicians based on the authors’ expertise and experience with health conditions of immigrants in detention. The survey was reviewed for clarity and understanding by clinicians who were not involved in the survey creation. The first survey question asked for the clinician to record their written consent to participate in the survey.

Health care professionals were surveyed regarding their demographics and practice characteristics, as well as their attitudes about the impact of detention on health and whether they ask patients if they have been detained. Clinicians were also asked if they treated patients who had been detained, and if so, to estimate their perceptions regarding the detrimental health effects of immigrant detention on their patients, the number of their patients who experienced adverse health effects due to poor conditions in detention and if they had reported cases to the authorities. In addition, clinicians were able to provide additional information regarding specific cases as free text.

Health care professionals were surveyed using both multiple choice and free text responses. The survey was divided into 4 sections: 1) clinicians’ demographics and practice characteristics, 2) clinicians’ attitudes and practices related to the impact of detention on health, 3) quantification of the number of patients that clinicians treated who experienced adverse health effects due to poor conditions in detention, characteristics of those patients and details of their cases, and, 4) clinicians’ experience and knowledge of how to report cases to authorities.

Clinicians estimated the number of patients treated and the types of illnesses for which they were treated. Simple sums of the estimates were used to calculate the total number of patients reported.

Multiple-choice questions were either single response, such as ‘Do you ask patients if they have been in detention?’ (Yes/Sometimes/No) or multiple responses, such as ‘Which languages do you speak with your patients?’ (English, Spanish, French, Haitian Creole, Other). In both cases, frequency and percentages were calculated using the number of clinicians responding as the denominator. For questions where providers could select more than one response, the sum of the percentages can be greater than 100%.

### Data collection

The survey was sent to listservs and professional email lists which the authors had access to, including Emergency Medicine, Pediatric, Family Medicine, and the Physicians for Human Rights (PHR) Asylum Network clinicians over the course of 2 months (October 1 – December 1, 2020). Repeat responses from the same IP address were not allowed. The clinicians on these listservs work extensively with immigrant populations and represented a key subset of clinicians who were likely to have treated patients who had previously been in immigration detention. The exact number of clinicians who received the survey is not known, but the PHR asylum list had 2022 clinicians at the time that the survey was disseminated. Some clinicians also belong to multiple listservs and may have received the invitation more than once (but repeat responses were not allowed).

### Data analysis

Frequency and percentages were calculated using providers/clinicians as the denominator. For questions where providers could select more than one response, the sum of the percentages could be greater than 100%. More rigorous statistical testing was not performed because we did not believe we were examining an unbiased population.

The survey was designed, distributed, and conducted online using Qualtrics software, [Qualtrics, Provo, UT, 2020]. SAS was used for data analysis [SAS Enterprise Guide V7.1, SAS Institute Inc., Cary, NC, 2017]. Tableau was used for data visualization [Tableau Desktop V2020.4, Tableau Software LLC, 2020, Seattle, WA]. This project was deemed exempt from Stanford University Human Research Protection Program institutional review board (Protocol 55,394 - Dr. Nancy E. Wang)) review due to the anonymity of both provider and patient.

## Results

### Demographics

**Fig. 1:**
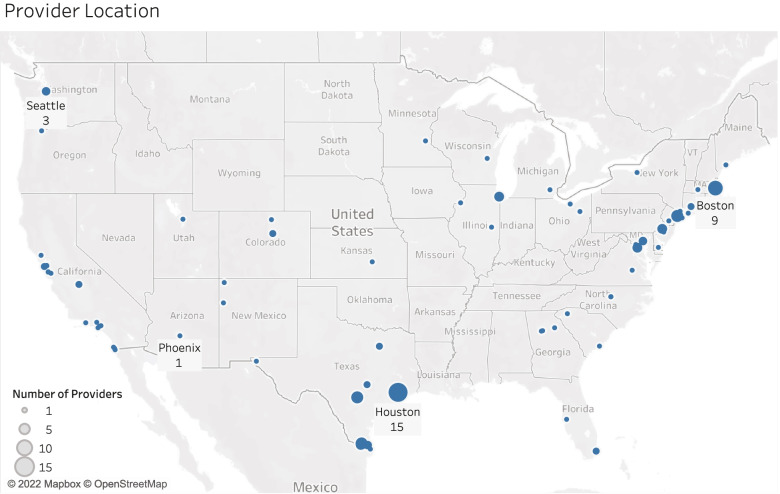
Location of health care providers. The size of the dot indicates the number of providers in the location

There were 150 responses received with complete practitioner demographics. Eighty-five, or approximately half of the respondents (57%), observed medical conditions they attributed to detention and included details about their observations. Of the 150 health care practitioners, just over 75% were physicians and another 15% were mental health professionals. Table 2 provides an overview of clinician characteristics. Practitioners worked throughout the United States (Fig. 1). The practitioners who did and did not observe medical conditions attributable to detention were similar, except that those who observed medical conditions related to detention were more likely to speak foreign languages and to not be located in the Northeast (Table 2).

**Table 2:** Characteristics of Clinicians answering survey

Provider Characteristics	All respondents (who completed demographic questions)	Providers who observed medical conditions relating to detention
**N**	**150 (100.0%)**	**85 (56.7%)**
Race/Ethnicity		
White	114 (76.0%)	67 (78.8%)
Hispanic	32 (21.3%)	21 (24.7%)
Asian or Pacific Islander	21 (14.0%)	11 (12.9%)
Native American	7 (4.7%)	5 (5.9%)
Black	6 (4.0%)	1 (1.2%)
Other	5 (3.3%)	3 (3.5%)
Sex		
Female	109 (72.7%)	64 (75.3%)
Male	41 (27.3%)	21 (24.7%)
Languages Spoken		
English-only	42 (28.0%)	14 (16.5%)
Spanish	95 (63.3%)	62 (72.9%)
French	11 (7.3%)	9 (10.6%)
Haitian Creole	4 (2.7%)	3 (3.5%)
Other	17 (11.3%)	8 (9.4%)
Years in Practice		
< 1	9 (6.0%)	2 (2.4%)
1–5	41 (27.3%)	25 (29.4%)
6–10	30 (20.0%)	18 (21.2%)
11–20	30 (20.0%)	19 (22.3%)
21–30	22 (14.7%)	14 (16.4%)
> 31	18 (12.0%)	7 (8.2%)
26–30	12 (8.0%)	7 (8.2%)
Type of Profession		
MD/DO	116 (77.3%)	65 (76.5%)
Mental Health Professional	22 (14.7%)	13 (15.3%)
NP/PA	6 (4.0%)	4 (4.7%)
Public Health Professional	6 (4.0%)	3 (3.5%)
Specialty^a^		
Pediatrics	59 (39.3%)	37 (43.5%)
OB/GYN	11 (7.3%)	7 (8.2%)
Family Medicine	18 (12.0%)	8 (9.4%)
Internal Medicine	17 (11.3%)	9 (10.6%)
Emergency Medicine	13 (8.7%)	9 (10.6%)
Mental Health	30 (20.0%)	17 (20.0%)
Other Specialty	21 (14.0%)	12 (14.1%)
Setting^a^		
Outpatient (non-urgent)	112 (74.7%)	64 (75.3%)
Urgent Care	17 (11.3%)	11 (12.9%)
Emergency Department	23 (15.3%)	14 (16.5%)
Inpatient	46 (30.7%)	25 (29.4%)
ICU (includes NICU, PICU)	18 (12.0%)	9 (10.6%)
Other Setting	10 (6.7%)	4 (4.7%)
Shelter/Legal	4 (2.7%)	3 (3.5%)
Institution^a^		
Academic	93 (62.0%)	54 (63.5%)
County/City Dept of Health	11 (7.3%)	7 (8.2%)
Federal Qualified Health Clinic	25 (16.7%)	20 (23.5%)
Private Practice	29 (19.3%)	15 (17.6%)
Other	24 (16.0%)	7 (8.2%)

^a^Practitioners could indicate all specialties, settings and institutions in which they practiced, thus these categories can add up to greater than 100%

### Survey responses to structured questions

The vast majority of health care practitioners surveyed (98%) believed that detention affected health (Table 3); although only 67 (44.7%) “routinely” and 44 (29.3%) “sometimes” asked if patients had been in detention. The major reasons for not always asking were: “I’m not sure how to frame the question” (24.0%) and “It’s not relevant to the patients I see” (26.0%).

**Table 3:** Clinicians’ attitudes regarding whether detention affects health and why they do not ask patients if they have been in detention

N	150
Do you believe detention affects health	
Yes	147 (98.0%)
No/Unsure	3 (2.0%)
Do you ask patients if they have been in detention	
Yes	67 (44.7%)
Sometimes	44 (29.3%)
No	39 (26.0%)
Reasons for not always asking^a^	85^b^
I’m not sure how to frame question in every situation	36 (42.4%)
It’s not relevant to the patients I see	35 (41.2%)
I don’t always have time	19 (22.4%)
It interferes with patient trust	16 (18.8%)
Do not think to	9 (10.6%)
Concern for/previous experience of patient (re)traumatization.	7 (8.2%)
Other	8 (9.4%)
No answer	2 (2.4%)

*N* = 85 because only the 85 physicians who completed the survey were asked this question

^a^Practitioners could indicate all reasons which pertained, thus these categories can add up to greater than 100%

The eighty-five clinicians who observed medical conditions attributed to detention reported a combined estimate of 1300 patients with a medical issue related to their time in detention (Table 4). Seventy-five (88%) clinicians observed patients with delayed access to medical care or medicine in detention, including vaccine preventable diseases, need for prenatal care, and medications which were taken away. Thirty-nine (46%) clinicians observed patients with new or acute health conditions including infection and injury they attributed to their time in detention; this included 36 (42%) of clinicians who saw patients with mental health symptoms. Fifty (59%) clinicians saw worsened chronic conditions or special needs conditions. Forty-five (53%) clinicians observed patients who delayed care after detention.

**Table 4:** Estimate of Reported Patients Experiencing Health conditions related to detention

Health Condition	Number and Percent of Estimated Patients	Number and Percent of Surveyed Providers^**a**^
**Total Patients with health conditions related to detention**	**1300 (100%)**	**85 (100%)**
**Delayed access or lack of access to appropriate medical care and medication**		75 (88.2%)
Patients with vaccine-preventable conditions acquired in detention (Varicella).	83 (6.4%)	17 (20.0%)
Patients whose medications were taken away or denied access to their medications during their time in detention.	307 (23.6%)	55 (64.7%)
Patients who required pre-natal, delivery and/or post-partum care during their time in detention	163 (12.5%)	26 (30.6%)
**New, acute health condition**		39 (45.9%)
Patients diagnosed with or experiencing symptoms consistent with COVID19 during detention or within 2 weeks of release from detention	84 (6.5%)	22 (25.9%)
Patients with non-COVID19 infections acquired during detention (GI, Respiratory, etc.)	169 (13.0%)	26 (30.6%)
Patients with injuries acquired during detention (musculoskeletal, burns)	78 (6.0%)	21 (24.7%)
Patients who were subjected to substandard living conditions that affected their health (malnutrition, dehydration)	241 (18.5%)	31 (36.5%)
Patients with mental health symptoms related to their time in detention (anxiety, depression, PTSD)	402 (30.9%)	36 (42.4%)
**Worsened chronic condition or special needs condition**		
Patients with chronic conditions that worsened during detention (diabetes, heart disease)	253 (19.5%)	50 (58.8%)
**Other concerning health issues**		
Patients with other concerning health circumstances not covered above	341 (26.2%)	61

^a^Practitioners could indicate all conditions seen, thus these categories can add up to greater than 100%

### Qualitative analysis of free text responses

Below we provide details of categories of medical issues with the largest quantity of comments in the free text boxes, namely lack of access to medications, mental health concerns, and lack of access to health care after discharge. Surveyed clinicians provided short descriptions of memorable cases they attributed to poor conditions and subpar medical care in detention. Table 5 highlights additional cases reported, categorized into the following themes: Pregnant women, Children, Mentally Ill, COVID-19, and Other.

**Table 5:** Clinicians’ recollection of Select Cases Involving Individuals recently released from immigration detention

Themes	Illustrative Descriptions
**Cases involving Pregnant people**	“Patient with pyelonephritis that went untreated while in detention center, was released only when she went into unstoppable preterm labor due to her infection”. “Patient was told by medical providers at the detention center that she was not pregnant, and thus was not provided with any prenatal care. Was released when she reached full term gestation, and ended up giving birth with her IUD still in place because no one at the detention facility would remove it for her”. “The one I constantly think about is a woman who was pregnant and kept complaining of stomach pain. She was told it was reflux and given tums. She complained several times and finally, a week after the pain started, was brought to the hospital. She was found to have an ectopic pregnancy- a pregnancy outside of the uterus, in one of her fallopian tubes. We took her back to the OR emergently and took out the ectopic pregnancy, but her entire belly was full of blood. She had clearly been bleeding for a while.” “A case of a young lady who was pregnant in the third trimester. Brought into the emergency department due to headaches, elevated blood pressures. Found to have severe range blood pressures along with other markers of pre-eclampsia with severe features and an intrauterine fetal demise”. “We had a third-trimester pregnant patient who was clearly visibly pregnant (and reported that she had advised authorities of her pregnant status) who had no basic health intake or blood pressure check, and despite complaining to authorities that she didn’t feel well she wasn’t taken for medical attention until she had an eclamptic seizure. She was critically ill from the time she was transported from the original hospital she was taken to (unequipped to handle the level of care she needed). She didn’t follow up as needed due to fear she would be taken back to the detention center.”
**Pediatric cases**	A child in family detention for 4 months who demonstrated malnutrition based on weight for stature in first percentile, and weight loss over the first 2 months of his detention. He was given inadequate diet and medical care during this period. Child with juvenile dermatomyositis whose prescription medications were confiscated and whose condition deteriorated because of lack of access to medications upon arrival in our community. Child with seizure disorder whose medications were confiscated and who was ultimately hospitalized. A 10 yr old with asthma, meds taken away while in detention and not returned, had asthma exacerbation after release and mother had no meds. Teenage boy with refractory epilepsy that was ultimately deemed surgically resectable (2 years after his arrival), who upon arrival had limited supply of Vimpat and was not provided with a bridge supply or adequate substitute while in detention. His second medicine, Keppra, was available. Teenager held in ORR shelter × 1 year, misdiagnosed bipolar, sedated on meds × 6 months and had PTSD, seen by psychiatry at discharge and taken off of these meds Child unnecessarily kept in detention despite the fact that his mother was available because staff reasoned she could not take care of his behavioral needs (including a form of selective mutism). Through my evaluation and interview with mother, I realized the minor was not cognitively impaired but traumatized. Child with undiagnosed congenital heart disease who came to clinic with dyspnea and oxygen saturation in the 70’s A minor who acquired an ankle fracture and was not treated for days. Infant with concern for dehydration separated from minor breastfeeding mom and given to adult dad. Neonate with fever and cyanosis. Dehydration from gastroenteritis. Severe respiratory infections and respiratory distress.
**Mental health poorly addressed**	I followed one schizophrenic male who was decompensating and put into solitary and treated with vistaril and antidepressants. It took close to a year to get him on an antipsychotic. Out of control dm II, depression with psychosis sent out with no housing, ptsd not diagnosed The staff were insensitive, took clothing away from the transgender woman which was particularly hurtful.
**Other serious health issues**	Case of patient placed on incorrect HIV regimen for months and experienced worsening resistance profile (which was already very severe) further limiting treatment options. HIV virus level never reached undetectable, but appropriate resistance testing never performed and regimen never changed. Patients with post-concussive syndrome getting no imaging or treatment with significant morbidity.
**COVID-19 related Care**	Young woman with COVID, tachy to 160 s documented, reported CP/SOB/palpitations. Detention center did not get any imaging, ECG, or labs (except for a routine thyroid study) and had no consideration of PE/MI/arrhythmia/etc. They sent her back to her cell with no vitals for 13 h and told her to “drink more water”. A 3 yo experienced constipation and poor weight gain as a result of inappropriate diet during a 3 month detention. He also got influenza and fractured a finger in a metal door at the facility. He was on COVID quarantine (22 h in a small room with his mother and brother) for 14 days following trip to ER for his finger A 40yo experienced worsening of severe depression, PTSD, and passive suicidality in ICE detention. He was afraid to report medical complaints (chest pain and flank pain with a medical history significant for prior ureteral obstruction) because he was afraid of the mental health suffering he would experience in medical isolation for COVID. A woman with Multiple chronic conditions ready for release and got COVID.

### Lack of access to medications

The theme of lack of access to medications was pervasive in most free text responses. Physicians reported that a large number of patients have been denied access to various medications, including medications to prevent seizures, asthma medications, blood pressure and heart failure medicines, insulin or other diabetes medications, antidepressants or antipsychotic medication, and HIV medications. Sometimes an alternative medication was provided but was inadequate, such as a clinician who reported a “low supply of anti-epileptic medications or inadequate substitute available within the center.” Two clinicians mentioned a lack of access to hormone treatment for gender-affirming care for transgender patients. Clinicians also reported specific cases including a patient with congenital hypothyroidism whose levothyroxine was taken away, patients with lupus juvenile dermatomyositis and glaucoma who did not receive their medications while in detention, and a patient suffering from psychosis (delusions) who relapsed due to a forced discontinuation of their psychotropics.

### Abuse and mental health conditions

Using free-text response, several clinicians noted that some patients reported abusive conditions in detention, including physical and sexual assault and verbal abuse: “Patients subjected to sexual assault and verbal and physical harassment”; “Traumatizing interactions or neglect with resulting prolonged emotional distress”; “Hunger strikes, being sprayed with tear gas in detention”; “People screamed at and demeaned by US border/detention officials” and “An indigenous child in a juvenile detention facility was tasered”. Given reports of abuse, it is perhaps not unsurprising that clinicians consistently noted the high prevalence of mental health issues among patients who had been in detention, and that they received inadequate treatment for post-traumatic stress disorder (PTSD), anxiety, and depression. Clinicians also reported their perception that the detention experience itself was linked with worsened psychological symptoms, observing: “severe emotional distress caused by being detained”; “Decompensation of pre-existing psychiatric conditions”; and “The experience of detention exacerbates PTSD and other mental health problems.”

### Access to health care after release from detention

Many of the clinicians reported that recently released individuals were often not able to access the health care that they needed post-release, primarily due to fear that accessing care would lead to tracking by immigration enforcement which would result in either return to detention or deportation. Most clinicians described fear of accessing care for chronic conditions or preventative care, but some clinicians also reported that even acutely ill patients were too afraid to access urgently needed treatment: “I had a patient who delayed seeking care despite having daily seizures for 2 weeks; he went into status epilepticus and was transported to the hospital and found to have a brain tumor”; “failure to show for outpatient epilepsy appointments at a time when ICE apprehensions in the community were increasing”; “Critically ill patient didn’t follow up after hospital discharge due to fear.”

A number of clinicians also indicated their perception that experiences in detention resulted in a high overall level of mistrust in the health care system’s ability or intent to safeguard patients’ well-being, as one clinician put it, “Most of them were wary of encountering the system”, while others described patient attitudes as “cautious”, “fearful” or “not comfortable”. A couple of clinicians noted that having experienced poor care or mistreatment in the past impacted immigrants’ sense of deservingness as patients, as one clinician said, “They don’t know their rights to access healthcare”; and another described, “This person experienced feelings of not deserving basic care because she was criminalized.” Tele-health was one modality which some patients felt more comfortable to access, as one clinician noted, “We started doing more prenatal care over the phone when ICE enforcement was expanded within the interior of the United States, because of patient concern about being detained again.”

### Clinician knowledge and practices regarding reporting to government authorities

Lastly, while clinicians reported caring for immigrants who had been detained in CBP, ICE and ORR custody, their responses to the structured questions indicated that 22% did not know in which agency their patients had been detained. When asked if they reported some of these concerning encounters to anyone, the vast majority did not. Reasons for not reporting included: “I did not know I could report” (43.6%); “I didn’t know why or how to report” (45.5%); The cases didn’t meet reporting criteria (25.5%); I didn’t want to bring attention/pressure on the patient (21.8%); and other (20.0%) (note that percentages add to greater than 100% because multiple options could be chosen). Reasons clinician did not report (written in the free text option) included: “Patient requested that I not report,” and “seems futile.” Of the 21 providers who reported, 3 reported to the local health department, 6 reported to the Department of Homeland security,2 reported to Child Protective services (CPS), and 13 reported to “other” agency including attorneys and advocates, institutional social workers, and client immigration lawyers.

## Discussion

In this unique inquiry into clinicians’ perceptions of the health effects of US immigrant detention, clinicians attributed acute or worsening medical conditions in their patients to delayed access to appropriate medical care, poor living conditions and lack of access to medications while in custody. Concerns regarding mental health conditions and access to care were particularly prevalent.

This is the first survey, to our knowledge, of health care professionals treating individuals upon release from detention. The results of this survey, although not a nationally representative sample, serve as a source of credible information about conditions experienced within immigration detention facilities and is a means of corroborating testimonials from immigrants themselves or from media reports due to the lack of transparency by federal entities, limited responses to Freedom of Information Act (FOIA) requests, and ethical and legal barriers to survey clinicians working within the system or detainees themselves.

The high proportion of reported mental health conditions within this case series, while not from a representative sample, is aligned with previous evidence of high rates of post-traumatic stress disorder (PTSD), anxiety, and an association of being in detention with deteriorating mental health outcomes even when controlling for prior trauma [15, 16]. The findings in this survey also remind us of the unique vulnerabilities of women and children in detention. These results, coupled with increased knowledge of the effects of toxic stress [17], specifically on children with adverse childhood experiences (ACES [18]), increase the urgency to reform immigration protocols that emphasize detention rather than community-based alternatives, to release individuals from immigration detention, to decrease the length of detention, and to improve the conditions of detention.

Reports about patients’ reluctance to seek care because of fear of Immigrations and Customs Enforcement (ICE) and deportation corroborate earlier studies as well [12, 19–23].

Immigration is a known and significant social determinant of health, as is immigration detention [24, 25]. There is a broad consensus among experts that being held in detention has a cumulative adverse effect on health [15, 26–29]. While attention is focused on reversing harmful policies, it is important to consider systemic changes to the immigration system at large.

Healthcare professionals have sought to address negative health consequences of detention in various ways. They have spoken up as whistleblowers in 2018 on the severe health risks at stake in forced family separation and family detention [30] and in 2020 on the lack of COVID-19 mitigation measures that put both detention facility staff and the detainees at risk [12, 31, 32]. Organizations such as Medical Review for Immigrants, Doctors for Camp Closure, and Physicians for Human Rights have engaged in medico-legal work to review medical records of detainees and to assist immigration attorneys seeking to obtain urgent humanitarian release for their clients with worsening serious medical conditions attributed to subpar medical care in detention facilities. Other clinicians who continue to work in these contexts, or with patients who are detained or recently released, may face moral distress or dual loyalty challenges [33]. The finding that clinicians who observed medical conditions related to detention were more likely to speak foreign languages and not be located in the Northeast reflects that patients may feel more comfortable talking about difficult experiences with clinicians who speak their language [34] and due to the concentration of detention facilities in the south and southwest of the United States [35]. A 2012 descriptive study in Massachusetts found that women clinicians and primary care physicians were more likely to notice negative impact of immigration enforcement on patients, but that was not reflected in our data [36].

Our study had several limitations. First, our survey respondents comprise a self-selected group, consisting of clinicians who work with immigrant patients and other marginalized populations, and routinely serve as advocates for social justice and equity in health. They are thus oriented and sensitized to explore and elevate systemic issues negatively affecting these populations. These factors may reflect both a selection and a perception bias. Second, we used a snowball sampling methodology rather than random sampling. As such, our clinician health care professional population, while distributed across geographical areas, specialties and practice settings, is not representative of the wider clinician community engaged with immigrant populations. This may contribute to an under-representation of health situations involving formerly detained individuals. Third, this survey is based on self-reporting and is thus subject to recall bias. We did not review medical records of individual patients, nor require any proof or validation of the situations reported by the survey respondents. Lastly, and importantly, we did not interview members of the population in question themselves. While the information included in this survey is second-hand and subject to various limitations as noted above, healthcare clinicians represent a highly credible professional group.

## Conclusions

Our survey assesses clinician perceptions about the adverse health impact of immigration detention on migrant populations based on real-life clinical encounters. These perceptions unfortunately corroborate other testimonials and media reporting of medical neglect and worsened mental and physical health in detention facilities. Our findings can help inform policy discussions specifically surrounding systematic changes to the delivery of healthcare in detention, quality assurance and transparent reporting, specifically for the medical community.

## Data Availability

The datasets analyzed during the current study are available from the corresponding author on reasonable request.

## References

[1] Alvarado M, Balcerzak A, Barchenger S, Campbell J, Carranza R, Clark M, et al. Deaths in custody. Sexual violence. Hunger strikes. What we uncovered inside ICE facilities across the US. USA Today 2019. Available from: https://www.usatoday.com/in-depth/news/nation/2019/12/19/ice-asylum-under-trump-exclusive-look-us-immigration-detention/4381404002/ [cited 2021 Apr 29]

[2] The Fatal Consequences of Dangerously Substandard Medical Care in Immigration Detention. In: Human Rights Watch. 2018. Available from: https://www.hrw.org/report/2018/06/20/code-red/fatal-consequences-dangerously-substandard-medical-care-immigration [cited 2021 Apr 29]

[3] Detained: How the US built the world's largest immigrant detention system. The Guardian. 2019. Available from: https://www.theguardian.com/us-news/2019/sep/24/detained-us-largest-immigrant-detention-trump [cited 2021 Apr 29]

[4] Immigration Detention in the United States by Agency. In: American Immigration Council. 2020. Available from: https://www.americanimmigrationcouncil.org/sites/default/files/research/immigration_detention_in_the_united_states_by_agency.pdf [cited 2021 Apr 29]

[5] FAQs (2021). Identification and Monitoring of Pregnant Detainees. ICE: U.S. Immigration and Customs Enforcement.

[6] American Immigration Lawyers Association (2019). Attorney General Barr Strips Bond Eligibility from Asylum Seekers: Matter of M-S- Analysis and Q&A. National Immigrant Justice Center.

[7] Health Risks of Customs and Border Protection Detention. In: Physicians for Human Rights. 2019. Available from: https://phr.org/wp-content/uploads/2019/07/PHR-Fact-Sheet_Health-Risks-of-CBP-Detention.pdf [cited 2021 Apr 29]

[8] Gassama H, Altman H, Tidwell Cullen T, Small M (2019). Toolkit Immigration Detention Oversight and Accountability. National Immigrant Justice Center.

[9] Immigration Detention 101 (2020). Detention Watch Network.

[10] Grassini M, Terp S, Fischer B (2021). Characteristics of deaths among individuals in US immigration and customs enforcement Detention facilities, 2011-2018. JAMA Netw Open.

[11] Parmar P, Ross M, Terp S, Kearl N, Fischer B, Grassini M, Ahmed S, Frenzen N, Burner E (2021). Mapping factors associated with deaths in immigration detention in the United States, 2011-2018: a thematic analysis. Lancet Region Health Am.

[12] Erfani P, Uppal N, Lee CH, Mishori R, Peeler KR (2021). COVID-19 testing and cases in immigration Detention centers, April-august 2020. JAMA..

[13] Peeler K, Erfani P, Lee CH, Uppal N, Hampton K, Raker E, Mishori R (2021). Praying for Hand Soap and Masks.

[14] Caitlin Patler, Altaf Saadi, Maria-Elena De Trinidad Young, Konrad Franco. Release from US immigration detention may improve physical and psychological stress and health: results from a two-wave panel study in California, SSM Mental Health. 2021;1, 10.1016/j.ssmmh.2021.100035. 100035, ISSN 2666-5603.

[15] von Werthern M, Robjant K, Chui Z, Schon R, Ottisova L, Mason C, Katona C (2018). The impact of immigration detention on mental health: a systematic review. BMC Psychiatry.

[16] Keller AS, Rosenfeld B, Trinh-Shevrin C, Meserve C, Sachs E, Leviss JA, Singer E, Smith H, Wilkinson J, Kim G, Allden K, Ford D (2003). Mental health of detained asylum seekers. Lancet..

[17] Shonkoff JP, Garner AS, Siegel BS (2012). The lifelong effects of early childhood adversity and toxic stress. Pediatrics..

[18] Felitti VJ, Anda RF, Nordenberg D, Williamson DF, Spitz AM, Edwards V, Koss MP, Marks JS (1998). Relationship of childhood abuse and household dysfunction to many of the leading causes of death in adults: The Adverse Childhood Experiences (ACE) Study. Am J Prev Med.

[19] Hacker K, Chu J, Leung C, Marra R, Pirie A, Brahimi M, English M, Beckmann J, Acevedo-Garcia D, Marlin RP (2011). The impact of immigration and customs enforcement on immigrant health: perceptions of immigrants in Everett, Massachusetts, USA. Soc Sci Med.

[20] Saadi A, Sanchez Molina U, Franco-Vasquez A, Inkelas M, Ryan GW (2020). Assessment of perspectives on health care system efforts to mitigate perceived risks among immigrants in the United States: a qualitative study. JAMA Netw Open.

[21] Rhodes SD, Mann L, Simán FM, Song E, Alonzo J, Downs M (2015). The impact of local immigration enforcement policies on the health of immigrant Hispanics/Latinos in the United States. Am J Public Health.

[22] Maldonado CZ, Rodriguez RM, Torres JR, Flores YS, Lovato LM (2013). Fear of discovery among Latino immigrants presenting to the emergency department. Acad Emerg Med.

[23] Lee J, Bruce J, Wang NE. Opportunities for supporting Latino immigrants in emergency and ambulatory care settings. J Community Health. 2020:1–8. 10.1007/s10900-020-00889-7 PMID: 32700173; PMCID: PMC7373833. 10.1007/s10900-020-00889-7 PMC7373833 32700173

[24] National Academies of Sciences, Engineering, and Medicine; Health and Medicine Division; Board on Population Health and Public Health Practice; Roundtable on the Promotion of Health Equity (2018). Immigration as a Social Determinant of Health: Proceedings of a Workshop.

[25] Gurrola MA, Ayón C (2018). Immigration policies and social determinants of health: is immigrants’ health at risk?. Race Soc Probl.

[26] Cleveland J, Kronick R, Gros H (2018). Symbolic violence and disempowerment as factors in the adverse impact of immigration detention on adult asylum seekers’ mental health. Int J Public Health.

[27] MacLean SA, Agyeman PO, Walther J, Singer EK, Baranowski KA, Katz CL (2019). Mental health of children held at a United States immigration detention center. Soc Sci Med.

[28] Saadi A, De Trinidad Young ME, Patler C, Estrada JL, Venters H (2020). Understanding US immigration Detention: reaffirming rights and addressing social-structural determinants of health. Health Hum Rights.

[29] Erfani P, Chin ET, Lee CH, Uppal N, Peeler KR (2021). Suicide rates of migrants in United States immigration detention (2010-2020). AIMS. Public Health.

[30] Miriam Jordan, Whistle-Blowers Say Detaining Migrant Families ‘Poses High Risk of Harm’, The New York Times, July 18, 2018. https://www.nytimes.com/2018/07/18/us/migrant-children-family-detention-doctors.html.

[31] McKenzie K, Mishori R (2020). Releasing migrants from detention during the Covid-19 pandemic. J Gen Intern Med.

[32] Mishori R. Risk behind bars: Coronavirus and immigration detention. The Hill. 2020. Available from: https://thehill.com/opinion/immigration/487986-risk-behind-bars-coronavirus-and-immigration-detention[cited 2021 Apr 29]

[33] Sirkin S, Hampton K, Mishori R (2021). Health professionals, human rights violations at the US-Mexico border, and holocaust legacy. AMA J Ethics.

[34] Al Shamsi H, Almutairi AG, Al Mashrafi S, Al KT. Implications of language barriers for healthcare: a systematic review. Oman Med J. 2020; Available from: https://www.ncbi.nlm.nih.gov/pmc/articles/PMC7201401/ [cited 2021 Nov 20]. 10.5001/omj.2020.40 PMC7201401 32411417

[35] Trac Immigration - Comprehensive, independent, and nonpartisan information about immigration enforcement. Available from: https://trac.syr.edu/immigration/quickfacts/. [cited 2021 Nov 20]

[36] Hacker C (2012). Arsenault and Marlin, Provider’s perspectives on the impact of immigration and customs enforcement (ICE) activity on immigrant health. J Health Care Poor Underserved.

